# The dirty business of eliminating open defecation: The effect of village sanitation on child height from field experiments in four countries

**DOI:** 10.1016/j.jdeveco.2022.102990

**Published:** 2022-11

**Authors:** Lisa Cameron, Paul Gertler, Manisha Shah, Maria Laura Alzua, Sebastian Martinez, Sumeet Patil

**Affiliations:** a University of Melbourne, Australia; b University of California Berkeley and NBER, USA; c UCLA, BREAD and NBER, USA; d CEDLAS-PEP and Facultad de Ciencias Económicas Universidad de La Plata, Argentina; e International Initiative for Impact Evaluation-3ie, USA; f NEERMAN, India

**Keywords:** I12, I15, O15

## Abstract

We examine the impacts of a sanitation program designed to eliminate open defecation in at-scale randomized field experiments in four countries: India, Indonesia, Mali, and Tanzania. The programs – all variants of the widely-used Community-Led Total Sanitation (CLTS) approach - increase village private sanitation coverage in all four locations by 7–39 percentage points. We use the experimentally-induced variation in access to sanitation to identify the causal relationship between village sanitation coverage and child height. We find evidence of threshold effects where increases in child health of 0.3 standard deviations are realized once village sanitation coverage reaches 50–75%. There do not appear to be further gains beyond this threshold. These results suggest that there are large health benefits to achieving coverage levels well below the 100% coverage pushed by the CLTS movement. Open defecation decreased in all countries through improved access to private sanitation facilities, and additionally through increased use of sanitation facilities in Mali who implemented the most intensive behavior change intervention.

## Highlights

• Examine impact of sanitation program in India, Indonesia, Mali, and Tanzania.
• Treatment increases village private sanitation coverage by 7–39 percentage points.
• Identify causal relationship between village sanitation coverage and child height.
• Child health gains realized once village sanitation coverage reaches 50 percent.
• Suggests large health benefits achieving coverage levels well below 100% coverage.

## 1 Introduction

Open defecation (OD) is a major cause of the persistent worldwide burden of diarrhea and enteric parasite infection among children under 5 years old (Mara et al., 2010). OD can lead to the fecal contamination of water and food supplies and to the transmission of soil-borne helminths (Chavasse et al., 1999). Reducing OD requires access to and use of improved sanitation facilities, typically toilets of some form, defined as facilities that prevent human feces from re-entering the environment.^2^ In 2015, 32% of the world population did not have access to even basic sanitation services (WHO-UNICEF, 2019). Given the large externalities associated with OD, families are only fully protected if both they and their neighbors have access to and use improved sanitation facilities. This has led to interventions that focus on the OD practices of the community, rather than solely of the household.

In this paper we report the results of at-scale cluster (village) randomized controlled trials of sanitation interventions in four countries: India, Indonesia, Mali, and Tanzania. The core of the intervention – *Community Led Total Sanitation* (CLTS) - is the same across all countries, with some variation in terms of intensity of treatment (greater intensity in Mali) and the inclusion of subsidies for toilet construction (India). The trials are at-scale in the sense that the interventions were implemented by governments as part of their national environmental health strategies and randomly rolled out geographically over time. The combination of at-scale cluster-randomized field experiments with common measurement of outcomes in four countries provides not only strong internal validity but also a degree of external validity not seen in most studies.^3^

Our study makes three important contributions. First, we find that treatment is, in all four countries, associated with a higher probability of a household having its own private improved sanitation facility (by 7–39 percentage points) and reduces self-reported open defecation by similar amounts in most cases. The interventions produced increases in village sanitation coverage with larger increases in countries that had lower baseline coverage (for example, on average by 18 and 29 percentage points in Mali and India respectively). This is consistent with the notion that last mile effects are the hardest due to diminishing marginal returns to the treatment.

Second, we examine the impacts of treatment on child health (height). Limited health impacts are found in India and Indonesia, consistent with recent experimental studies that have found positive effects of CLTS on sanitation facilities and open defecation but limited effects on child health outcomes (Cameron et al., 2019; Pickering et al., 2019; Null et al., 2018; Luby et al., 2018; Briceño et al., 2017; Patil et al., 2014). In Mali, however, the most intensive intervention site, we find children in treated villages are 0.16 standard deviations taller on average than in control villages.

One potential reason for the limited effects on child health is that individual treatment effects mask heterogeneity derived from the externalities associated with community coverage. What matters is the amount of open defecation in your community and not just by your own household. Therefore, an individual household that installs sanitation facilities may have no impact on health if few of their neighbors have sanitation facilities. Significant empirical relationships between sanitation coverage and child height have been found in India using non-experimental methods (Augsburg and Rodriguez-Lemes, 2018; Spears, 2020). Almost all previous field experiments, however, ignore such heterogeneity except Cameron et al. (2021) which finds relatively large effects on height from greater community sanitation coverage in Laos.

We use the experimentally induced variation in village sanitation coverage to identify the causal relationship between community sanitation coverage and child height. We exclude Tanzania where we do not have information on baseline child height. However, because we have three countries with varying amounts of community coverage, we are able to investigate this issue by exploiting the experimental variation treatment induces in sanitation coverage. In a linear specification, we estimate that going from no coverage to 100 percent coverage yields a 0.43 standard deviation increase in child height.^4^

When we examine nonlinearities in the relationship between village sanitation coverage and height, we find evidence of threshold effects. After village sanitation coverage reaches 50 percent, large benefits to child height of 0.30 standard deviations accrue. This child height effect size persists until the village achieves near-universal coverage. These results suggest that there are large health benefits to achieving around 50 percent coverage, but limited effects to higher coverage rates. This finding challenges the stated aim of CLTS of achieving 100 percent Open Defecation Free (ODF) communities. It appears health gains may be realized with considerably lower sanitation coverage than 100 percent.

The third contribution is to examine the mechanisms driving the impacts. We examine the extent to which the construction of latrines and behavior change that does not accompany construction play a role in reducing OD. In Indonesia, India, and Tanzania, the health promotion campaigns worked primarily through getting households to invest in in-home private sanitation facilities that lower the cost of using a toilet. Investment in sanitation was also important in Mali, but there it was also accompanied by behavioral change in the form of greater use of facilities that existed at baseline. In Mali, CLTS treatment reduced open defecation both among those who had sanitation facilities at baseline and among those who did not. Only the behavioral change aspects of the CLTS intervention can explain the reduction in open defecation among those who already had sanitation at baseline. Hence CLTS can work through both sanitation construction and behavioral change if the behavioral change component is strong enough.

## 2 Interventions

CLTS is one of the most popular sanitation interventions worldwide, having been implemented in more than 60 countries throughout Asia, Latin America, and Sub-Saharan Africa.^5^ CLTS programs are large-scale, community-targeted and community-driven campaigns designed to promote and improve sanitation practices in rural areas (Kar and Chambers, 2008). CLTS seeks to harness social pressure through community meetings in which the negative health consequences of open defecation for the community are discussed and communities are encouraged to develop plans and commit to becoming 100 percent open defecation free (Kar and Pasteur 2005; Kar and Chambers, 2008). Facilitators are sent to villages for a few days to lead graphic discussions of the community's current sanitation practices, the health consequences of such practices, and to facilitate collective action plans to eliminate open defecation. The facilitated discussions are held in public places and are open to all community members. They involve a “walk of shame,” where villagers are asked to provide a tour indicating where people defecate. A map of the village is drawn on the ground and villagers are asked to indicate where they live, where they defecate, and the routes they take there and back. The facilitator then helps people analyze how fecal contamination is spreading from the exposed excreta to their living environments and food and drinking water. It becomes apparent that everyone is ingesting small amounts of each other's feces. The premise underlying the program's approach is that this process prompts feelings of disgust that lead to personal and collective desire to solve the problem with the ultimate aim of becoming an Open Defecation Free community.^6^ CLTS is intended to be participatory in nature and facilitates communities to take a decisive role in ensuring that each member internalizes the implication of open defecation (Sah and Negussie, 2008). The community is then on its own to forge a plan of action with, at best, limited support from the program.

One of the key aspects of CLTS is its encouragement of households to build and use sanitation facilities that prevent fecal matter from re-entering the environment and inhibit flies from transmitting pathogens from the fecal matter to food and water that are later ingested. While CLTS-derived solutions could involve building shared toilets or public toilets, in practice the main outcome has been the construction of private in-home, water-flushed squat toilets with drainage to a sealed pit. Households and communities are typically left to their own devices to finance and implement the construction of these facilities as CLTS by itself does not provide resources for this purpose. CLTS aims to produce completely open defecation free communities.

While CLTS was the common intervention, as discussed above, there were a number of differences across the four countries (See Table 1). India's Total Sanitation Campaign supplemented traditional CLTS with monetary subsidies to households for the construction of private in-home sanitation facilities. The amount of the Indian subsidy depends on whether a household was defined to be Below the Poverty Line (BPL) or Above the Poverty Line (APL). The program provided materials and cash of Rs 4200 (US $84) to BPL households and Rs 2000 (US $40) to non-BPL households to support toilet construction. This was intended to cover the cost of building a complete toilet.

**Table 1:** Intervention design, experimental design and data.

	India	Indonesia	Mali	Tanzania
A. Intervention Design				
Geographic Location	2 rural Districts in Madhya Pradesh	8 rural Districts in East Java	Province of Koulikoro	10 rural Districts all over country
# CLTS Visits to Communities	One CLTS visit	One CLTS visit & one follow-up visit to reinforce messages	One CLTS visit &12 follow-up visits over a year to reinforce messages	One CLTS visit & one follow-up visit to reinforce messages
Subsidy for construction	Yes	No	No	No
B. Experimental Design				
Random Assignment	yes	yes	yes	yes
Unit of Assignment	Village	Village	Village	Village
Stratification	Block	Subdistrict	None	District
Treatment Group Compliance	100%	66%	98%	84%
Control Group Contamination	25%	14%	10%	0%
Average Exposure period	6 months	24 months	18 months	23 months
C. Data				
Date Baseline Survey	May–July 2009	Aug–Sept 2008	April–July 2011	None
Date Endline Survey	Feb–April 2011	Nov 2010–Jan 2011	April–June 2013	May–Dec 2012
Number of Villages	80	160	121	90
Number of Households	1655	1908	7461	1800
Number of Children under 5	2046	2300	6745	N/A
Treatment Attrition Rate	7.9%	4.4%	6.1%	N/A
Control Attrition Rate	7.4%	4.1%	6.4%	N/A
D. Descriptive Statistics (Control Group Means)				
Private Household Sanitation	0.121	0.52	0.342	0.37
Village Sanitation Coverage	13.1%	51.0%	34.2%	50%
Height-for-Age Z scores	−1.85	−1.72	−1.3	–

Notes: In all four countries, we explore the impact of Community-Led Total Sanitation (CLTS) interventions. Panel A presents the geographic location and the intensity of the CLTS visits. In Mali, facilitators visited communities first for CLTS triggering and then monthly for one year to reinforce CLTS messaging. In Indonesia and Tanzania, facilitators visited the communities only twice for a triggering visit with a second follow-up visit to reinforce CLTS messaging. India only had one visit for triggering and no follow-up. India supplemented CLTS with monetary subsidies to poor households for the construction of private in-home sanitation facilities. Panel B shows the experimental design. In all four countries, a cluster-randomized intervention at the village level was implemented. In 3 out of the 4 countries (Indonesia, India, and Tanzania), the villages were first clustered into strata, and then the villages were randomized into treatment and control groups within each stratum. Panel C presents the timeline for the data collection, sample size, and attrition levels for each country. Detailed information on the interventions and experimental designs from the individual countries can be found in Patil et al. (2013 and 2014) for India; for Indonesia, see Cameron and Shah (2010) and Cameron et al. (2013). For Mali see Alzua et al. (2014). For Tanzania see Briceño et al. (2017). Panel D presents baseline means for the control group for India, Mali and Indonesia. Control group means at endline are reported for Tanzania as there was no baseline survey conducted in Tanzania.

Further, there were substantial differences in the intensity of the CLTS interventions. In Mali, facilitators visited communities first for CLTS triggering and then monthly for one year to monitor activities and reinforce CLTS messaging. In contrast, in Indonesia and Tanzania facilitators visited the communities only twice, once for a triggering visit with a second follow-up visit shortly thereafter to reinforce CLTS messaging. India had the lightest CLTS intensity with only one visit for triggering and no systematic follow-up.

**Fig. 1:**
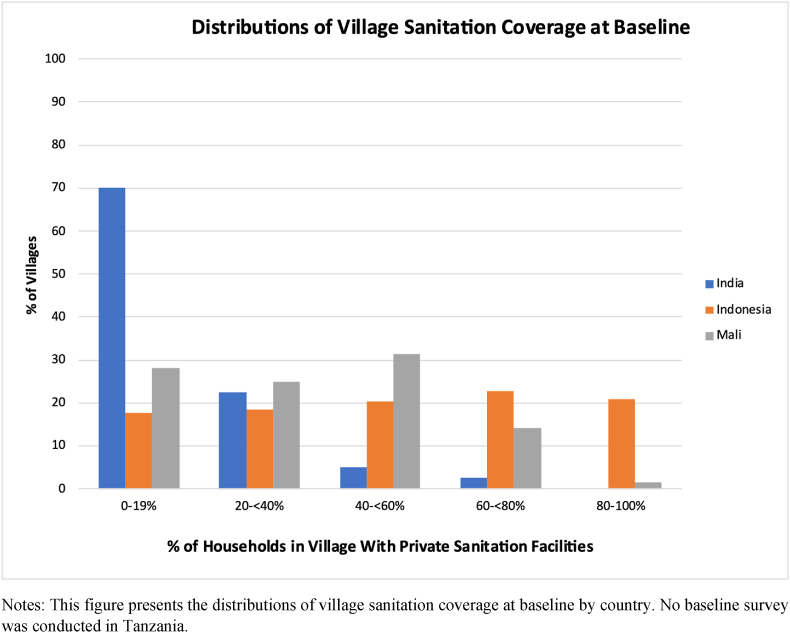
Distribution of Village Sanitation Coveage at Baseline. Notes: This figure presents the distributions of village sanitation coverage at baseline by country. No baseline survey was conducted in Tanzania.

The context also differs across the various trials. Mali is the poorest setting, followed by Tanzania and India, with Indonesia being the most prosperous. This ranking also holds in terms of their human development indices, and for the provinces in which the trials were conducted.^7^ There is also substantial heterogeneity in baseline village-level sanitation. Indonesia has the highest sanitation coverage at baseline at 51 percent. India, however, rather than Mali, has the lowest baseline sanitation coverage at 13 percent, with most villages having less than 20 percent of households with private sanitation (Fig. 1). Mali's sanitation coverage at baseline was 34 percent. No baseline survey was conducted in Tanzania but coverage in control villages was relatively high at 50 percent.

The details of the random assignment and data collection are summarized in Table 1 and discussed in detail in the data appendix. In all four countries, random samples of households with children under two (at baseline) were surveyed. In general, the samples for all four countries are well balanced at baseline (see Appendix Tables A1 – A4), have low levels of attrition, and show little evidence of attrition bias. More detailed information on the individual interventions, experimental designs, complete balance tests, and findings from the individual country impact evaluations can be found in Cameron and Shah (2010) and Cameron et al. (2013) for Indonesia, in Patil et al. (2013) for India, in Alzua et al. (2014) for Mali, and in Briceño et al. (2017) for Tanzania.

Compliance with the experimental design was not perfect (Table 1). In Indonesia, only 66 percent of the villages assigned to treatment were triggered through CLTS activities (compliance), while 14 percent assigned to the control group also received the intervention (contamination). Similarly, 25 percent of the villages in India and 10 percent in Mali assigned to the control group received treatment. Non-compliance with the evaluation design likely reflects the capacity of the local implementing governments. In fact, Mathematica (2011) found that program implementation varied significantly across districts in Indonesia, reflecting differing implementation capacity of local government and cross-sectoral commitment to the program. In the analyses below, we estimate intention-to-treat regressions, comparing the outcomes of the group assigned to treatment to the group assigned to control.

## 3 Access to sanitation

### 3.1 Household access to sanitation

We commence by examining the impact of the program on households’ access to sanitation to test whether treatment increased sanitation coverage. We estimate the following regression specification for the sample of households who did not have private in-home sanitation facilities at baseline:

(1) $$S_{ijk}=\alpha +\beta T_{jk}+\sum_{k}{\gamma_{k}R}_{k}{+\varepsilon }_{ij}$$

where $S_{ijk}$ takes on the value one if household *i* in village *j* in randomization strata *k* has access to sanitation facilities at endline, $T_{jk}$ takes on the value one if village *j* in randomization strata *k* was assigned to treatment, and $R_{k}$ takes on the value one if village *j* was in stratum *k*.

We consider three sanitation outcomes, including access (i) to any sanitation facilities, (ii) to private in-home facilities, and (iii) to shared or public facilities outside the home. The estimates in (1) are identified off the random assignment and are intention-to-treat (ITT) parameters. The standard errors are clustered at the village level.

The first column of Table 2 reports estimates of the impact of treatment on access to any sanitation facilities. We see statistically significant positive effects in all four countries. The largest impact is in Mali where sanitation access increased by 39 percentage points, an increase of 267 percent over the control group. The next highest impact is in India where access increased by 24 percentage points, an increase of 169 percent over the control group. The impacts of treatment on access in Indonesia and Tanzania are more modest, amounting to 47 and 19 percent increases over control group, respectively. Except in Indonesia, all of the large increases in access to sanitation come through construction of private in-home sanitation facilities. In Indonesia, about half of the increase in sanitation access comes from expanded access to shared out-of-home facilities. The effectiveness of the Mali intervention is underscored by the fact that this is the poorest context and previous studies have shown that it is more difficult to get poorer households to build toilets due to resource constraints (see Cameron et al., 2019). Table A5 in the appendix reports results where we interact treatment with the percent of the village population living in poverty. The interaction term is negative and statistically significant for any sanitation in Indonesia but not statistically significant in India, Tanzania, or Mali.

**Table 2:** Impact of treatment on access to sanitation facilities, among households without private sanitation facilities at baseline.

	(1)	(2)	(3)
	Any Sanitation	Private Sanitation	Shared Sanitation
Indonesia			
Treatment	0.076*** [0.023]	0.043** [0.017]	0.034* [0.018]
Sample Size	937	937	937
Control Mean	0.163	0.081	0.095
India			
Treatment	0.238*** [0.037]	0.236*** [0.034]	0.002 [0.006]
Sample Size	1453	1453	1453
Control Mean	0.141	0.133	0.008
Mali			
Treatment	0.390*** [0.029]	0.381*** [0.029]	0.009** [0.004]
Sample Size	2632	2632	2632
Control Mean	0.146	0.141	0.005
Tanzania			
Treatment	0.134*** [0.034]	0.153*** [0.029]	−0.019 [0.027]
Sample Size	1323	1323	1323
Control Mean	0.702	0.372	0.330

Notes: This table reports the estimated effect of treatment on the probability that the household has access to any sanitation facility (column 1), a private facility on their property (column 2), and a shared public or private facility not on their property (column 3). Each treatment effect comes from a separate estimation of equation (1) for the outcome specified at the start of each column. Each panel corresponds to a different sample (country). Columns 1–3 include randomization strata fixed effects. Robust standard errors, clustered at the village level, are presented in brackets below the treatment effect coefficients. ***p < 0.01, **p < 0.05, *p < 0.1.

### 3.2 Village sanitation coverage

A household's protection from the pathogens spread through open defecation depends on both their own behavior and the behavior of their neighbors. Eliminating their own open defecation will have limited protection if their neighbors continue to practice open defecation. In the next set of analyses we regress village sanitation coverage (the proportion of households sampled in the village who have private sanitation at endline) on treatment status and baseline private sanitation coverage.^8^ As the effect of treatment on village sanitation coverage will likely vary depending on village sanitation coverage at baseline, we include its interactions with treatment status. As is shown in Fig. 1 there is substantial heterogeneity both within and between countries in baseline village-level sanitation rates. As a baseline survey was not conducted in Tanzania, we drop it from this analysis.

**Fig. 2:**
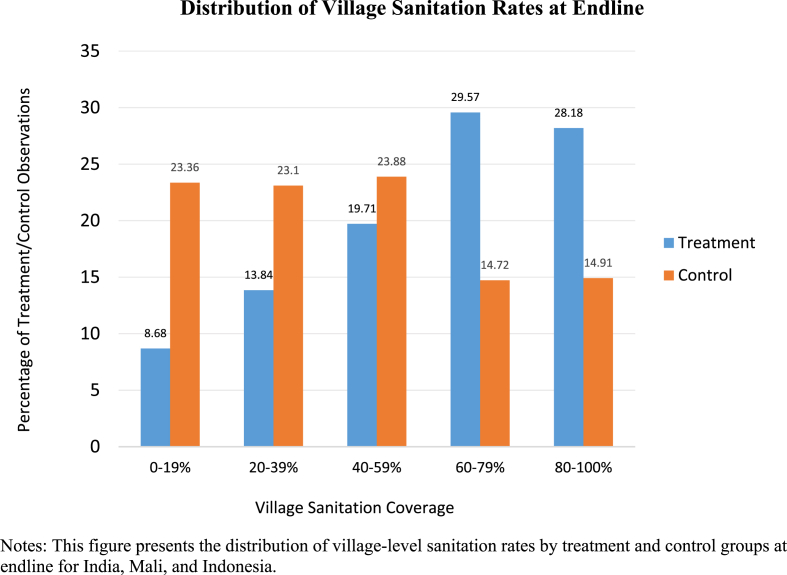
Distribution of Village Sanitation Rates at Endline. Notes: This figure presents the distribution of village-level sanitation rates by treatment and control groups at endline for India, Mali, and Indonesia.

Fig. 2 presents the distributions of village-level sanitation rates by treatment and control groups at endline. Treatment is associated with a clear shift from the lowest sanitation quintiles to the higher quintiles. Twenty-three percent of control villages had less than twenty percent sanitation coverage compared to less than nine percent of treatment villages at endline. Whereas 28 percent of treatment villages attained sanitation coverage in excess of 80 percent, compared to 15 percent of control villages.

Table 3 presents the estimation results for the impact of treatment on village sanitation coverage. We estimate the following equation:

(2) $$S_{jk}=\alpha +\beta T_{jk}{+S_{jk}^{BL}+S_{jk}^{BL}*T_{jk}+\sum_{k}{\gamma_{k}R}_{k}+u}_{jk}$$

where $S_{jk}$ is the share of households sampled in village *j* in randomization strata *k* which have private sanitation at endline, $S_{jk}^{BL}$ is defined analogously for the situation at baseline, and the other variables are as defined above.

**Table 3:** Impact of Treatment on Share of Households that have Sanitation at Endline (at village level).

	(1)	(2)	(3)	(4)
	Indonesia	India	Mali	Pooled
Treatment	0.040 [0.035]	0.274*** [0.047]	0.421*** [0.054]	0.232*** [0.028]
BL Coverage	0.809*** [0.059]	0.903*** [0.172]	0.731*** [0.085]	0.955*** [0.038]
BL Coverage x Treatment	−0.007 [0.059]	−0.686*** [0.250]	−0.389*** [0.124]	−0.205*** [0.051]
Sample Size	160	80	121	361
Control Mean	0.617	0.226	0.393	0.459

Notes: This table presents the estimation results for the impact of treatment on village sanitation coverage at endline from equation (2). Columns 1–3 include randomization strata fixed effects. The pooled estimates in column 4 include country-fixed effects. Robust standard errors are reported in brackets below the treatment effects. ***p < 0.01, **p < 0.05, *p < 0.1.

The results confirm the patterns observed in Fig. 2. Endline sanitation coverage increases as a result of treatment in both India and Mali. The coefficient on the interaction between baseline coverage and treatment is also negative and strongly significant for India and Mali, indicating that the impact of treatment on village sanitation coverage is smaller in villages that had higher coverage at baseline. For villages with no sanitation coverage at baseline, it is estimated that village sanitation increased by 27.4 and 42.1 percentage points in India and Mali respectively. At mean baseline village sanitation coverage for each country, the increases associated with treatment were estimated to be 18.4 percentage points in India and 28.8 percentage points in Mali. In Indonesia, while the coefficient is positive, there is no statistically significant impact of treatment on village sanitation coverage. (Baseline village sanitation coverage was 13.1 percent in India, 34.2 percent in Mali, and 51 percent in Indonesia.) The interaction between treatment and baseline sanitation coverage is negative but small and not statistically significant for Indonesia. The pooled estimates (which control for country context and include country fixed effects) show that treatment increased village sanitation on average across the countries and that an additional ten percentage points of coverage at baseline reduced the impact of treatment on endline sanitation coverage by approximately 2 percentage points.

## 4 Child health

We now assess the extent to which the CLTS treatment improved child health outcomes measured by the height of children who were less than two years old at baseline, an age at which height is sensitive to parasitic infections, diarrhea, and illness in general. We construct height-for-age z-scores, which place the child's height in the distribution of a well-nourished reference population for her age. We use a standardized age- and gender-specific growth reference based on WHO standards (2006, 2007).

Our empirical approach is based on the health capital model originally proposed in Grossman (1972) that specifies health as a stock that accumulates as a function of investment:

(3) $$H_{t}=I_{t}+\left(1-\delta \right)H_{t-1}+\varepsilon_{t}$$

In (3) $H_{t}$ is the stock of health capital in period *t*, $I_{t}$ is investment in health capital such as nutrition, prevention and curative medical care, and prevention activities such as exercise, safe water and sanitation; δ is the depreciation rate, and $\varepsilon_{t}$ is a shock to health in period *t*.

### 4.1 Reduced form treatment effects

We first estimate the average treatment effects of the interventions using a version of equation (3) where the investment is reduced open defecation, which is generated by the interventions. We estimate the following equation:

(4) $$H_{ijt}=\alpha +\beta T_{jt}+\gamma H_{ijt-1}+\varepsilon_{ijt}$$

where $T_{jt}$ takes on the value one if village *j* received the intervention in period *t*. In this case, β is the ITT estimate of the impact of treatment on child height. By conditioning on lagged $H_{t-1}$, β is interpreted as the effect of village sanitation coverage on child growth between the two periods.

Table 4 presents the results. We exclude Tanzania as we do not have data on baseline child height. Average child height is below the WHO reference group mean (i.e. the mean height-for-age z-score is negative) in all three countries for which we run the estimations. The average child in the control village in India (Mali; Indonesia) is 2.4 (1.75; 1.6) standard deviations shorter than the average child in the WHO reference group.^9^

**Table 4:** Impact of treatment on child height for age Z-score.

	(1)	(2)	(3)
	Indonesia	India	Mali
Treatment	−0.038 [0.034]	0.038 [0.118]	0.194** [0.078]
Sample Size	1869	1286	2418
Control Mean	−1.646	−2.384	−1.754
Control St. Dev.	1.054	1.532	1.355

Notes: This table reports the estimated effect of treatment on the height for age z-scores. This table presents coefficients from estimating equation (4). Each treatment effect comes from a separate linear regression (by country). All models include controls for baseline z-score and randomization strata fixed effects. Robust standard errors are clustered at the village level and are reported in brackets below the treatment effects. ***p < 0.01, **p < 0.05, *p < 0.1.

The estimated average treatment effects on height in India and Indonesia are small and not statistically significant, whereas in Mali the estimated effect size is 0.16 standard deviations and is statistically significant. The effect in Mali may be due to the more intensive nature of the program there. These results are consistent with the mixed estimates of treatment effects of CLTS-based interventions on child height in the literature.^10^

### 4.2 Effect of village sanitation coverage on child height

One potential reason for the limited reduced form treatment effects of CLTS on child height is that individual treatment effects mask heterogeneity derived from the externalities associated with community coverage. What matters is the amount of open defecation in your community and not just by your own household.

We estimate the causal relationship between village sanitation coverage and child height by exploiting the experimentally induced variation in village sanitation coverage for identification. To do so, we pool the data from the three countries for which we have data on child height both at baseline and follow-up: India, Indonesia and Mali. Since both households that did and did not have access to sanitation at baseline may benefit from the cleaner community environment, we do not restrict the sample to households with no access to sanitation at baseline. The analysis sample includes 5481 observations from 361 villages.

The general approach will be to replace $I_{t}$ from equation (3) with measures of village levels of sanitation and estimate an equation of the following form:

(5) $$H_{ijt}=\alpha +\beta S_{jt}+\gamma H_{ijt-1}+\varepsilon_{ijt}$$

where $S_{jt}$ is the sanitation coverage rate in village *j* in period *t*. Again, by conditioning on lagged $H_{t-1}$, β is interpreted as the effect of village sanitation coverage on child growth between the two periods.

We estimate (5) using standard OLS (column 1, Table 5) and by IV LASSO (columns 2–4, Table 5) using the data pooled across countries.^11^ We implement the IV strategy using the machine learning post-regularization procedure for linear models with multiple controls and instruments proposed by Chernozhukov et al. (2015) and Ahrens et al. (2018). This procedure is often referred to as post-double selection. The core of the procedure consists of using a LASSO regression with data-driven penalty loadings to obtain a sparse set of controls and instruments that allow for valid inference about the endogenous regressors and a sparse set of instruments that approximates that of the optimal instrument. The set of instruments includes the assignment of treatment at the village level and baseline village sanitation coverage by country, country dummies and all interactions. The potential set of additional controls includes individual and household characteristics measured at baseline.

**Table 5:** Effect of Village Sanitation on Child Height for Age z-scores.

	(1)	(2)	(3)	(4)
	OLS	IV-LASSO with PDS-selected variables and full regressor set		
Countries:	All	All	No Mali	Mali
Village Sanitation Coverage	0.301** [0.130]	0.474*** [0.117]	0.428*** [0.104	0.463*
				[0.239]
Sample Size (individuals)	5481	5481	3067	2418
Sample Size (villages)	361	361	240	121
Control Mean	−1.818	−1.818	−1.885	−1.729
Control St. Dev.	1.290	1.290	1.236	1.355
Kleibergen-Paap Wald rk F statistic		345.90	382.92	138.55

Notes: This table reports the estimated effect of village sanitation coverage on child height for age z-scores from equation (5). Results are estimated using pooled samples of children under 5 at baseline for India, Indonesia, and Mali. Column 1 reports results from the OLS specification and columns 2–4 report results from the IV Lasso specification. The IV regression uses the option PDS-selected variables and full regressor available in the LASSO command. The instruments for village sanitation coverage are: treatment in each country, sanitation coverage at baseline at the village level, and sanitation coverage at baseline interacted with treatment and country dummies. Column 2 selects the following instruments: treatment in Mali, sanitation coverage at baseline at the village level, and sanitation coverage at baseline interacted with treatment and an Indonesia dummy. Column 3 selects the following instruments: treatment in India, sanitation coverage at baseline at the village level, and sanitation coverage at baseline triple interacted with treatment and an India dummy. Column 4 selects the following instruments: treatment in Mali, sanitation coverage at baseline at the village level, and sanitation coverage at baseline triple interacted with treatment and a Mali dummy. Column 1 includes controls for baseline height of the child, country, an indicator of the randomization block, and a further set of controls all measured at baseline for each country interacted with a country dummy. The Indonesia controls include child age and sex dummies, education of the household head, household size, household per capita income, dirt floor, village is within a 10-min walk from a river, and discount rate. The India controls for controls include age and sex dummies, improved water source, hand washing station with soap and water, caregiver had correct knowledge about diarrhea, caregiver had correct knowledge about risks of open defecation, age and sex of household head, household size, and household income. The Mali controls include child age and sex dummies, education and language spoken by the household head, OD disapproval, asset index, and social capital index. The lasso-selected controls vary in columns 2–4. Robust standard errors are clustered at the village level and are reported in brackets below the main effects. ***p < 0.01, **p < 0.05, *p < 0.1.

Our approach assumes that treatment assignment affects child height only through village-level sanitation adoption and not through other channels such as changes to hygiene behavior such as purifying water or hand washing. In other words, the exclusion restriction assumes that treatment only affects child height via its effect on sanitation, not through other forms of behavioral change. Appendix Table A6 shows no treatment effects on hand-washing and water treatment behavior in Indonesia, Tanzania, or India. We do however find significant positive impacts on hand-washing in Mali (consistent with the more intensive behavioral change component in Mali, which we discuss further in Section 4 below). For this reason, we include additional results for a specification excluding Mali. The individual and household level controls are entered as interactions with indicator variables for the country in which the child lives. The results are reported in Table 5. The estimated effects are statistically significant for all of the models. Standard errors are clustered at the village level.

The impacts on child height reported in Table 5 are large enough to be meaningful. The estimated slope in the model excluding Mali (Column 3) indicates that full sanitation coverage in a village where no one previously has sanitation is associated with a 0.43 standard deviation increase in height. Another way to interpret the results is that a one standard deviation increase in village sanitation coverage (0.29) would yield about a 0.12 standard deviation increase in height.^12^

The results for Mali only (Column 4) show larger height gains with increases in village sanitation coverage than in the other countries (consistent with the program not just building toilets but being more effective in getting people to use them). Going from zero to total village sanitation coverage is estimated to increase height by 0.46 standard deviations in Mali. These results however need to be treated with caution given the concerns about the exclusion restrictions in this context.

We reject weak instruments using both the Stock-Yogo weak identification test critical values as well as Kleibergen-Paap F-statistics. The Kleibergen-Paap F-statistics from the first stages are large (see Table 5) and the instruments are selected from randomized treatment assignment status of the village interacted with country dummies, baseline village sanitation coverage, and baseline village sanitation coverage interacted with treatment status and country dummies. The notes in Table 5 describe which of these instruments are selected in each column by the IV Lasso procedure.

### 4.3 Nonlinearities

Table 6 further examines the form of the relationship between village sanitation coverage and child height. We allow the impact of village sanitation coverage on height to vary with the extent of coverage by including indicator variables for quartiles of village sanitation coverage (with 0–25 percent being the omitted category). We use IV LASSO with the instruments being 1) interactions between treatment status and country dummies; 2) indicator variables for quartile of baseline village sanitation coverage 3) treatment status interacted with the indicator of quartiles of baseline village sanitation coverage; and 4) triple interactions between treatment status the indicators of baseline sanitation coverage and country dummies. We use the same set of potential controls as in Table 5.

**Table 6:** Nonlinear Effects of Village Sanitation Coverage on Child Height for Age z-scores.

	(1)	(2)	(3)	(4)
	OLS	IV-LASSO with PDS-selected variables and full regressor set		
	All	All	No Mali	Mali
Panel A:				
Village Sanitation Coverage 25-49	−0.020 [0.088]	−0.008 [0.188]	−0.017 [0.210]	0.184 [0.391]
Village Sanitation Coverage 50-74	0.162* [0.087]	0.416*** [0.146]	0.259** [0.130]	0.572* [0.298]
Village Sanitation Coverage 75-100	0.202** [0.088]	0.251* [0.142]	0.220 [0.136]	0.412 [0.333]
Cragg-Donald Wald F statistic		42.26	41.12	48.28
Panel B:				
Village Sanitation Coverage 50-100	.187*** [0.071]	0.377*** [0.084]	0.297*** [0.084]	0.394*** [0.151]
Kleibergen-Paap Wald rk F statistic		105.92	148.00	81.34
Sample Size (individuals)	5481	5481	3063	2418
Sample Size (villages)	361	361	240	121
Control Mean	−1.818	−1.818	−1.885	−1.729
Control St. Dev.	1.290	1.290	1.236	1.355

Notes: This table reports the estimated effect of village sanitation coverage on child height for age z-scores. Results are estimated using pooled samples of children under 5 at baseline for India, Indonesia, and Mali. We allow the impact of treatment to vary with baseline sanitation coverage by including indicator variables for quintiles of village sanitation coverage (with 0–24% being the omitted variable in Panel A). Column 1 reports results from the OLS specification and columns 2–4 report results from the IV LASSO specifications. The set of instruments for village sanitation coverage are: treatment in each country, sanitation coverage at baseline at the village level (quartiles), and sanitation coverage at baseline (quartiles) interacted with treatment and country dummies. Column 2 selects the following instruments for Panel B: treatment in Mali, sanitation coverage at basline at the village level (quartiles), and sanitation coverage at baseline (quartiles) interacted with treatment and Indonesia country dummy. Column 3 selects the following instruments for Panel B: sanitation coverage at basline at the village level (quartiles). Column 4 uses the following instruments for Panel B: treatment in Mali, sanitation coverage at basline at the village level (quartiles), and sanitation coverage at baseline (quartiles) interacted with treatment and Mali country dummy. Column 1 includes controls for baseline height of the child, country, and an indicator of the randomization block and includes a separate set of controls all measured at baseline for each country interacted with a country dummy. The Indonesia controls include child age and sex dummies, education of the household head, household size, household per capita income, dirt floor, village is within a 10-min walk from a river, and discount rate. The India controls include age and sex dummies, improved water source, hand washing station with soap and water, caregiver had correct knowledge about diarrhea, caregiver had correct knowledge about risks of open defecation, age and sex of household head, household size, and household income. The Mali controls include child age and sex dummies, education and language spoken by the household head, OD disapproval, asset index, and social capital index. The lasso-selected controls vary in columns 2–4. Robust standard errors are clustered at the village level and are reported in brackets below the main effects. ***p < 0.01, **p < 0.05, *p < 0.1.

Table 6 reports results for all countries (OLS and IV) in columns 1–2, countries other than Mali (Indonesia and India) in column 3, and just Mali in column 4. All IV specifications show significant increases in height associated with sanitation coverage in excess of 50 percent. Height gains are not apparent below this level of coverage. This is consistent with there being a threshold effect - health gains are only realized once sanitation coverage has exceeded the threshold of, in this case, 50 percent. There appears to be no further gain when sanitation coverage extends beyond 75 percent. The coefficient on the indicator for sanitation coverage in the range of 75–100 percent is similar in magnitude to, and not significantly different from the 50–74 percent range. The same pattern is observed for all countries and the results excluding Mali. Panel B presents results where we constrain the impact of sanitation coverage in the 50–74 percent range to be the same as that for coverage in the 75–100 percent range. The results excluding Mali (Column 3) show that village sanitation coverage in excess of 50 percent is associated with a 0.30 standard deviation increase in height. Again, the results for Mali show larger impacts (0.39 standard deviation increase in height) but are suggestive given the potential violation of the exclusion restriction for Mali.^13^ We reject weak instruments using both the Stock-Yogo weak identification test critical values as well as Cragg-Donald Wald F statistics in Panel A when we have multiple endogenous variables (see Sanderson and Windmeijer, 2016) and Kleibergen-Paap F-statistics in Panel B.

The results in Table 6 have different implications for each of the countries depending on baseline village sanitation coverage. The biggest potential gains are in India where average baseline village sanitation coverage was about 13 percent and almost all villages had baseline coverage below 50% (see Fig. 1). This implies that almost all villages would experience significant and meaningful increase in child height if village sanitation coverage increased to above 50%. The lowest potential gains would be in Indonesia where about half the villages have already achieved well above 50% coverage. The potential in Mali is somewhere in between India and Indonesia (baseline village sanitation coverage was 34%). An important implication of the threshold results is that expanding village coverage beyond about 50% has limited impacts on child height so that countries with low levels of coverage have the most to gain.

## 5 Program mechanisms

In this section we lay out a framework that allows us to examine the contributions of behavioral and investment pathways. In order to separate out behavioral change pathways from investment pathways, we move our focus from household access to sanitation to open defecation.

We begin by noting that the interventions differentially affect those households that have and those that do not have existing private in-home sanitation facilities. For households who have existing private sanitation facilities in their house at baseline, the only pathway is through behavioral change, i.e. increased use of those facilities. In the case of families who do not have existing private in-home sanitation, an intervention can increase the use of shared (public or private) facilities outside the house or cause households to invest in private in-home sanitation. The investment in private sanitation facilities reduces the time and hassle or “transaction” cost of using sanitation facilities, thereby increasing use of sanitation facilities.

We formalize this discussion as follows. Let π(OD) be the probability of open defecation and π(S) be the probability of having private in-home sanitation facilities. Then the probability of open defecation can be written as the weighted sum of the conditional OD probabilities of those with and without private in-home sanitation facilities:

(6) π(OD)=π(OD|S=1)π(S)+π(OD|S=0)[1−π(S)]

In (6), π(OD|S=1) is the probability of OD conditional on having private in-home sanitation facilities and π(OD|S=0) is the probability of OD conditional on not having private in-home sanitation facilities.

To identify these components, we estimate the following regression for all households, and for households that have existing private in-home sanitation facilities at baseline respectively:

(7) $${OD}_{ijk}=\alpha +\beta T_{jk}+\sum_{k}{\gamma_{k}R}_{k}{+\varepsilon }_{ij}$$

where ${OD}_{ijk}$ is the OD rate of household *i* in village *j* in randomization strata *k* and the other variables are as defined previously. We cluster the standard errors at the village level.

The parameters in (7) are identified off the random assignment using the endline data.^14^ The dependent variable is an intensity measure of open defecation.^15^ The household was asked separately for men, women, and children if they defecated in the open always, sometimes, or never. We coded the answers 2 for always, 1 for sometimes, and 0 for never. We then summed the answers for the 3 types of household members. The values ranged from 0 to 6. We then divided by 6 in order to obtain a measure of OD intensity between 0 and 1, where 0 indicates no open defecation and 1 indicates always open defecation.

Table 7 presents the estimates of the impact of the program on households’ defecation behavior for Indonesia, India, Mali, and Tanzania. The first column reports estimates of the impact of treatment on open defecation for all households. We find negative effects in all four countries, of which three are statistically significant at conventional levels. As was the case for sanitation, the largest treatment effects on OD are in Mali where CLTS nudging was the most intensive. There we find that the OD rate fell by 0.33, which, when compared to the control group means, amounts to a 58 percent reduction in overall OD. Next highest is Tanzania where OD rates fell by 0.13, a 54 percent reduction in OD compared to the control group. The relative effects in Mali and Tanzania are about the same because the non-treatment OD rate in Tanzania (0.23) is less than half of that in Mali (0.57). In India and Indonesia, the effects sizes are substantially smaller at 10 percent or less.

**Table 7:** Impact of treatment on open defecation.

	(1)	(2)	(3)
	Full Sample	Households with Private Sanitation at Baseline	Households without Private Sanitation at Baseline
Indonesia			
Treatment	−0.019 [0.026]	0.005 [0.014]	−0.077*** [0.029]
Sample Size	1903	966	937
Control Mean	0.407	0.086	0.760
Control St. Dev.	0.457	0.197	0.397
India			
Treatment	−0.090*** [0.031]	−0.029 [0.042]	−0.091*** [0.020]
Sample Size	1655	202	1453
Control Mean	0.859	0.177	0.947
Control St. Dev.	0.320	0.252	0.198
Mali			
Treatment	−0.328*** [0.036]	−0.211*** [0.023]	−0.385*** [0.041]
Sample Size	3981	1383	2598
Control Mean	0.568	0.365	0.679
Control St. Dev.	0.374	0.318	0.355
Tanzania			
Treatment	−0.125*** [0.028]	−0.007 [0.006]	−0.135*** [0.034]
Sample Size	1786	467	1319
Control Mean	0.233	0.005	0.299
Control St. Dev.	0.423	0.070	0.458

Notes: This table reports the estimated effect of treatment on the household's degree of open defecation, and each treatment effect comes from a separate estimation of equation (7). Column 1 reports estimates of the impact of treatment on open defecation for all households. Column 2 presents the results for the sample of households who had private in-home sanitation at baseline. Column 3 reports estimates for those households that did not have private in-home sanitation facilities at baseline. Each panel represents a different sample (country). Columns 1–3 include randomization strata fixed effects. Robust standard errors are clustered at the village level and are reported in brackets below the treatment effects. ***p < 0.01, **p < 0.05, *p < 0.1.

In the second column we report the results for the sample of households who had private in-home sanitation at baseline. Here we are looking to see whether we observe any pure behavioral change (occurring in households that already had sanitation). In three out of the four countries, there was effectively no impact of treatment on the OD rates of households that had existing in-home private sanitation faculties. In Indonesia and Tanzania, this effect is most likely driven by the very low OD rates among these households to begin with. However, the OD rates among households with existing private sanitation are nontrivial in India and Mali. While treatment had a large negative effect on this group in Mali, it had no impact in India. This is consistent with the more intensive behavioral change intervention in Mali compared to India and explains the larger height gains associated with improvements in village sanitation coverage in Mali (Table 4), as the intervention caused households to build toilets and was more effective at getting household members to use them.

In the last column we report estimates for those households that did not have private in-home sanitation facilities at baseline. These estimates are a combination of the investment effect of the program (latrine construction) and the increased use of shared non-private sanitation facilities among those who chose not to construct. The results are interesting because, except for Mali, almost all of the overall reduction in OD comes from these households. In all countries, the estimated treatment effects are substantially larger than those for households with existing private in-home sanitation facilities. The treatment effect on households with no private sanitation at baseline is a reduction in open defecation by 7.7 ppts (10 percent) in Indonesia, 9.1 ppts (10 percent) in India, 38.5 ppts (57 percent) in Mali, and 13.5 ppts (45 percent) in Tanzania. The results in column 3 are analogous to the results in Table 2 for sanitation and show similar patterns. In the case of India, the estimated impacts on open defecation are much smaller than the effects on sanitation, possibly reflecting less use of the sanitation facilities than in the other countries.

In summary, only in Mali was there a significant behavioral change among those who had private in-home sanitation facilities. This suggests that a more intensive behavior change component induces people to use their existing facilities and stop defecating in the open. Augsburg et al. (2021) similarly find that continued follow up-activities were necessary to ensure continued safe sanitation in challenging locations (poor public infrastructure and low-quality sanitation facilities) in rural Pakistan. Without an intensive behavior change component, decreases in open defecation are largely a byproduct of the construction of sanitation facilities and, our results suggest, insufficient by themselves to promote child health.

## 6 Discussion

We examine the effects and mechanisms of CLTS sanitation promotion campaigns designed to eliminate open defecation in at-scale randomized field experiments in four countries: India, Indonesia, Mali, and Tanzania. The field experiments are at-scale in the sense that the interventions were designed and implemented by governments as part of their national environmental health strategies and randomly rolled out geographically over time. The combination of at-scale randomized field experiments in four countries provides not only strong internal validity but also a degree of external validity not seen in most studies.

The CLTS programs increased household access to sanitation in all four countries – by 267 percent in Mali, 169 percent in India, 47 percent in Indonesia and 19 percent in Tanzania. Mostly this was in the form of private, in-home sanitation. Despite the interventions increasing sanitation coverage, it does not appear that they were, on average, strong enough to individually be able to improve child health by reducing village-level OD. Only the Mali intervention is significantly associated with a 0.16 standard deviation increase in height. One reason for the limited reduced form treatment effects of CLTS on child height is that individual treatment effects mask heterogeneity derived from the externalities associated with community coverage. What matters is the amount of open defecation in your community and not just by your own household.

We estimate the causal relationship between village sanitation coverage and child height exploiting the experimentally induced variation in village sanitation coverage for identification. Estimation of the relationship between village sanitation coverage and child height, using the pooled data and the experimentally induced variation to identify the causal relationship, suggests that going from zero to 100 percent sanitation coverage results in an increase in height-for-age of at least 0.43 standard deviations.

We also find significant nonlinearities in the relationship between height and village sanitation coverage. There appear to be no gains in height for coverage below 50 percent, but large gains once the village reaches 50 to 75 percent coverage and limited gains at best beyond this level. This is consistent with there being a threshold effect - health gains are only realized once sanitation coverage has exceeded the threshold of, in this case, 50 percent. There appears to be no further gain when sanitation coverage extends beyond 75 percent.

These results have different implications for each of the countries depending on baseline village sanitation coverage. The biggest potential gains are in India where average baseline village sanitation coverage was about 13 percent and almost all villages had baseline coverage below 50 percent. This implies that almost all villages would experience significant and meaningful increase in child height if village sanitation coverage increased to above 50 percent. The lowest potential gains would be in Indonesia where about half the villages have already achieved well above 50 percent coverage. The potential in Mali is somewhere in between India and Indonesia.

An important implication of the threshold results is that expanding village coverage beyond about 50–75 percent has limited impacts on child height so that countries with low levels of coverage have the most to gain. These results suggest that there are large health benefits to achieving around 50 to 75 percent coverage which is a much lower level than the 100 percent pushed by the CLTS movement. Moreover, significant gains in health can be achieved can be gained without incurring the significant expenses associated with achieving costly last mile village coverage levels.

We also examine whether the programs worked through investment in sanitation facilities that lower the marginal cost of good sanitary behavior or through behavioral change resulting in increased use of existing sanitation facilities. The results address whether subsidies for health products are enough or whether nudges to use health products are necessary to change behavior sufficiently to improve health outcomes. Subsidies would be sufficient if households are simply liquidity constrained and have been unable to save enough or borrow to be able to build toilets. However, if open defecation is a deep-rooted habit that is culturally acceptable, then simply encouraging people to build toilets may not be enough to get people to use them. We find evidence that in Mali – where the behavioral change element of the program was strongest - a large portion of the reduction in open defecation came through behavioral change, i.e., increased use of existing sanitation facilities. Mali is also where we found the largest health impacts.

Overall, a substantial increase in the use of sanitation facilities among those who have access to such facilities combined with a large expansion and use of new sanitation facilities (possibly assisted by subsidies) was able to generate sufficiently large reductions in village OD to achieve meaningful improvements in health outcomes in Mali. Whether this approach is cost-effective in a wider range of contexts depends in large part on the price elasticity of the demand for sanitation facilities.

## Author statement

Cameron, Gertler and Shah share first co-authorship and led the analysis and drafting of this paper with support from the other authors. Cameron: Conceptualization, Methodology, Writing, Software, Project administration in Indonesia; Gertler: Conceptualization, Methodology, Writing, Software, Project administration, Funding acquisition; Shah: Conceptualization, Methodology Writing, Software, Project administration in Indonesia, Funding acquisition, corresponding author; Alzua: Software, Funding acquisition, Project administration in Mali; Martinez: Software, Funding acquisition, Project administration in Tanzania; Patil: Software, Funding acquisition, Project administration in India.

## Figures and Tables

**Table A1:** INDONESIA BALANCE TABLE

Variables	With Sanitation at BL			No Sanitation at BL			All Households		
	Mean (Treat)	Mean (Control)	p-value	Mean (Treat)	Mean (Control)	p-value	Mean (Treat)	Mean (Control)	p-value
Height-for-Age Z-score for children under 5	−1.282	−1.152	0.452	−1.642	−1.524	0.474	−1.467	−1.332	0.260
Weight for children under 5	8.36	8.28	0.561	8.173	8.28	0.377	8.263	8.281	0.840
Household has private sanitation facility	1	1	–	0	0	–	0.491	0.522	0.176
Household knows causes of diarrhea	0.159	0.135	0.287	0.091	0.079	0.506	0.124	0.108	0.265
Household has good OD knowledge	0.648	0.630	0.567	0.452	0.453	0.989	0.540	0.555	0.511
Household size	5.030	5.018	0.898	4.820	4.626	0.035	4.923	4.831	0.162
Age of household head	41.39	41.80	0.567	39.697	38.974	0.304	40.53	40.45	0.875
Male household head	0.933	0.946	0.422	0.959	0.976	0.139	0.946	0.960	0.151
Child is male	0.485	0.515	0.371	0.515	0.513	0.955	0.500	0.514	0.559
Household head completed primary	0.426	0.376	0.117	0.602	0.598	0.904	0.515	0.482	0.148
Household head completed secondary	0.206	0.233	0.314	0.193	0.189	0.878	0.200	0.212	0.497
Household head completed higher-secondary	0.316	0.348	0.294	0.137	0.138	0.946	0.225	0.248	0.239
Log per capita income	14.70	14.70	0.973	14.16	14.15	0.939	14.42	14.44	0.724
Household is poor	0.146	0.121	0.269	0.348	0.367	0.554	0.251	0.239	0.574
Household has dirt floor	0.146	0.185	0.106	0.320	0.343	0.448	0.234	0.261	0.188
River runs through village	0.690	0.640	0.098	0.782	0.796	0.615	0.737	0.714	0.266
Risk tolerant	0.185	0.142	0.031	0.160	0.170	0.644	0.172	0.155	0.244
Discount rate	0.599	0.584	0.360	0.609	0.601	0.652	0.604	0.592	0.321

Notes: This table presents baseline means for the Indonesia sample. Columns 1–3 show the means for the control group, the treatment group, and the difference between them for households with sanitation at baseline. Columns 4–6 show the means and differences between them for households without sanitation at baseline. Finally, columns 7–9 report the same means for all households.

**Table A2:** INDIA BALANCE TABLES

Variables	With Sanitation at BL			No Sanitation at BL			All Households		
	Mean (Treat)	Mean (Control)	p-value	Mean (Treat)	Mean (Control)	p-value	Mean (Treat)	Mean (Control)	p-value
Child had diarrhea in last 7 days	0.126	0.085	0.378	0.134	0.126	0.686	0.133	0.121	0.481
Mean Height-for-Age Z-score for under 5 children	−0.919	−1.414	0.005	−1.469	−1.917	0.048	−1.383	−1.848	0.014
Household reports OD as main sanitation option	0.0	0.0		0.940	0.891	0.309	0.796	0.774	0.602
% of Household in the village having improved sanitation facility	0.287	0.305	0.378	0.112	0.096	0.592	0.139	0.123	0.735
Household reports having improved sanitation facility	1.0	1.0		0.0	0.0		0.152	0.131	0.631
Household reports having improved drinking water source	0.984	0.936	0.069	0.884	0.762	0.018	0.898	0.785	0.010
Household with soap and water at their hand-washing station	0.920	0.963	0.095	0.386	0.508	0.013	0.467	0.568	0.016
Age in months of the child under 5 years	21.09	21.05	0.136	21.94	22.29	0.270	21.83	22.13	0.257
Age of household Head	51.42	51.33	0.841	45.129	42.040	0.002	46.10	43.26	0.003
Male household head	0.944	0.963	0.852	0.937	0.957	0.079	0.938	0.958	0.098
Household head attended school	0.880	0.840	0.181	0.453	0.509	0.149	0.519	0.553	0.318
Household head completed secondary school	0.632	0.575	0.104	0.303	0.338	0.436	0.354	0.369	0.741
Primary care giver knows the causes of diarrhea	0.707	0.716	0.585	0.668	0.638	0.294	0.674	0.648	0.296
Household belongs to schedule caste/tribe	0.330	0.223	0.063	0.725	0.778	0.104	0.668	0.705	0.314
House construction is robust (*pucca*)	0.888	0.917	0.062	0.534	0.558	0.532	0.588	0.605	0.530
Per capita monthly household income in Rupees	2962	3121	0.718	1551	1514	0.737	1767	1724	0.790
Household belongs to below poverty line category	0.248	0.193	0.333	0.351	0.427	0.012	0.336	0.396	0.044
Wealth Index (Principal Component based)	3.45	3.72	0.436	−0.60	−0.71	0.462	0.01	−0.13	0.698

Notes: This table presents baseline means for the India sample of household panel surveyed at both baseline and endline (n = 1655). Columns 1–3 show the means for the control group, the treatment group, and the statistical significance of the difference between them for households with private sanitation facility at baseline. Columns 4–6 and Columns 7–9 show these results for the households without private sanitation facility at baseline and for all households, respectively.

**Table A3:** MALI BALANCE TABLE

Variables	With Sanitation at BL			No Sanitation at BL			All Households		
	Mean (Treat)	Mean (Control)	p-value	Mean (Treat)	Mean (Control)	p-value	Mean (Treat)	Mean (Control)	p-value
Mean Height-for-Age Z-score for under 5 children	−1.177	−1.176	0.993	−1.185	−1.206	0.852	−1.180	−1.195	0.856
Mean Weight-for-Age Z-score for under 5 children	- 1.347	−1.375	0.770	1.323	−1.370	0.659	−1.339	−1.372	0.656
Age in months of the child under 5 years	18.680	18.742	0.9283	17.246	17.930	0.286	18.162	18.220	0.900
Household reports OD as sanitation option	0.913	0.8739	0.0582	0.964	0.952	0.430	0.929	0.916	0.406
OD is Not Acceptable in Community	0.746	0.808	0.0513	0.684	0.732	0.199	0.723	0.761	0.192
Household size (self-reported)	8.362	9.007	0.0193	6.392	7.024	0.006	7.677	7.564	0.668
Household is poor (lowest p (25))	0.068	0.034	0.133	0.145	0.152	0.863	0.098	0.112	0.651
Literacy (household head)	0.382	0.331	0.132	0.2584	0.277	0.614	0.306	0.314	0.803
Male household head	0.971	0.973	0.780	0.959	0.962	0.788	0.967	0.966	0.927
Age of household head	46.430	47.965	0.973	41.709	41.709	0.003	43.702	43.879	0.787

Notes: This table presents baseline means for the Mali sample. Columns 1–3 show the means for the control group, the treatment group, and the difference between them for households with sanitation at baseline. Columns 4–6 show the means and differences between them for households without sanitation at baseline. Finally, columns 7–9 report the same means for all households.

**Table A4:** TANZANIA BALANCE TABLE (ENDLINE DATA WITH RETROSPECTIVE VARIABLES)

Variables	With Sanitation at BL			No Sanitation at BL			All Households		
	Mean (Treat)	Mean (Control)	p-value	Mean (Treat)	Mean (Control)	p-value	Mean (Treat)	Mean (Control)	p-value
Clean lighting energy (electricity, solar, gas)	0.049	0.068	0.380	0.051	0.048	0.964	0.050	0.052	0.815
Electricity as main lighting energy source	0.019	0.044	0.268	0.031	0.024	0.441	0.027	0.028	0.842
Floor of main living area made of cement	0.209	0.223	0.293	0.121	0.128	0.992	0.147	0.149	0.970
Floor of main living area made of earth/clay	0.783	0.752	0.159	0.868	0.846	0.445	0.842	0.825	0.495
Male	0.867	0.883	0.913	0.894	0.858	0.134	0.886	0.864	0.205
Age	42.544	41.583	0.867	39.206	39.385	0.602	40.209	39.878	0.794
Can read and write	0.787	0.752	0.124	0.739	0.694	0.172	0.753	0.707	0.074
Years of Education (if attended school)	5.221	5.170	0.306	5.062	4.812	0.325	5.110	4.892	0.260
Muslim	0.373	0.320	0.780	0.279	0.277	0.115	0.307	0.286	0.082
Christian	0.578	0.655	0.662	0.634	0.584	0.780	0.617	0.600	0.889
HH size	5.354	5.024	0.472	4.856	4.841	0.940	5.006	4.882	0.437
Age of child when first cared by caregivers	0.087	0.053	0.155	0.092	0.083	0.573	0.090	0.076	0.448
Well main source of drinking water	0.304	0.388	0.180	0.319	0.365	0.322	0.314	0.370	0.195
Surface water main source of drinking water	0.388	0.354	0.546	0.376	0.441	0.197	0.379	0.422	0.439
HH treats their water	0.338	0.354	0.821	0.392	0.310	0.026	0.376	0.320	0.064
Owns another house	0.186	0.155	0.539	0.157	0.136	0.529	0.166	0.141	0.228

Notes: Tanzania does not have baseline data so this table presents means from endline data with retrospective questions. Columns 1–3 show the means for the control group, the treatment group, and the difference between them for households with sanitation at baseline. Columns 4–6 show the means and differences between them for households without sanitation at baseline. Finally, columns 7–9 report the same means for all households.

**Table A5:** Impact of Treatment on Access to Sanitation Facilities, Among Households Without Private Sanitation Facilities at Baseline, interacting treatment with Percentage of Poor Households at Baseline

	(1)	(2)	(3)
	Any Sanitation	Private Sanitation	Shared Sanitation
Indonesia			
Treatment	0.134*** (0.045)	0.054 (0.038)	0.088** (0.039)
Treatment*% Poor	−0.179 (0.141)	−0.015 (0.105)	−0.211 (0.136)
% Poor	−0.077*** (0.029)	−0.065*** (0.020)	−0.014 (0.023)
Sample Size	1087	1087	1087
Control Mean	0.173	0.089	0.100
India			
Treatment	0.160 (0.096)	0.154* (0.089)	0.006 (0.015)
Treatment*% Poor	0.226 (0.257)	0.232 (0.233)	−0.006 (0.043)
% Poor	0.038 (0.188)	−0.001 (0.162)	0.038 (0.039)
Sample Size	1453	1453	1453
Control Mean	0.141	0.133	0.008
Mali			
Treatment	0.406*** [0.030]	0.403*** [0.029]	0.003 [0.003]
Treatment*% Poor	−0.042 [0.037]	−0.066* [0.038]	0.024* [0.013]
% Poor	−0.109*** [0.020]	−0.105*** [0.020]	−0.004 [0.005]
Sample Size	2591	2591	2591
Control Mean	0.148	0.143	0.005
Tanzania			
Treatment	−0.017 (0.074)	0.094 (0.077)	−0.111 (0.072)
Treatment*% Poor	0.255 (0.162)	0.103 (0.154)	0.152 (0.130)
% Poor	−0.485*** (0.115)	−0.158* (0.095)	−0.327*** (0.109)
Sample Size	1323	1323	1323
Control Mean	0.702	0.372	0.330

Notes: This table reports the estimated effect of treatment on the probability that the household has access to any sanitation facility, a private facility on their property, and a shared public or private facility not on their property from equation (1). Each panel represents a different sample (country) and each column a different specification. Each treatment effect comes from a separate linear regression. Indonesia regressions include sub-district fixed effects, India block fixed effects, and Tanzania district fixed effects. Robust standard errors are clustered at the village level in Indonesia, India, Mali, and Tanzania; these are reported in brackets below the treatment effects. The definitions of poor are as follows: Indonesia – households in the bottom quartile of the distribution of non-land assets; India –households which have a below-poverty-line (BPL) ration card; Mali – households that fall below the asset index; Tanzania – households falling in the bottom half in terms of an asset-based wealth index. ***p < 0.01, **p < 0.05, *p < 0.1.

**Table A6:** Impact of Treatment on Hygiene and Water Practices

	(1)	(2)	(3)
	Hand-washing Behavior	Improved drinking water source	Effective drinking water treatment
Indonesia			
Treatment	−0.006 (0.005)	−0.011 (0.013)	0.017 (0.020)
Romano-Wolf p-value	0.570	0.781	0.781
Sample Size	1900	1903	1903
Control Mean	0.988	0.891	0.564
India			
Treatment	0.008 (0.007)	0.016 (0.013)	0.006 (0.004)
Romano-Wolf p-value	0.207	0.171	0.171
Sample Size	1655	1655	1655
Control Mean	0.981	0.954	0.005
Mali			
Treatment	0.174*** (0.031)	0.005 (0.056)	−0.004 (0.006)
Romano-Wolf p-value	0.004	0.944	0.351
Sample Size	5672	5672	5672
Control Mean	0.421	0.364	0.0105
Tanzania			
Treatment	0.003 (0.021)	−0.065 (0.042)	0.016 (0.023)
Romano-Wolf p-value	0.920	0.402	0.833
Sample Size	1792	1780	1792
Control Mean	0.257	0.337	0.225

Notes: This table reports the estimated effect of treatment on each of the dependent variables. *Hand-washing behavior* is in India and Indonesia a self-report of whether household members ever wash their hands after defecation, and in Tanzania is a self-report of washing hands with soap in the last 24 h; *Improved Drinking Water Source* equals 1 if the household reports the main source of drinking water being piped water, a public tap or standpipe, a tube well or borehole, a protected well, a protected spring, rainwater or bottled water, and 0 otherwise, in Tanzania it also includes water delivered by truck; *Effective drinking water treatment* is an indicator of whether the household reported treating its drinking water in the following ways: Indonesia, Mali and Tanzania – boiling; India - boiling, use of chemicals, electrical or candle filters. Each panel represents a different sample and each column a different specification. Each treatment effect comes from a separate linear regression. Indonesia regressions include sub-district fixed effects, India block fixed effects, Mali district fixed effects, and Tanzania district fixed effects. Robust standard errors are clustered at the village level in Indonesia, India, Mali, and Tanzania; these are reported in brackets below the treatment effects. ***p < 0.01, **p < 0.05, *p < 0.1.

**Table A7:** Threshold Effects of Village Sanitation Coverage on Child Height for Age z-scores. Using triple interaction terms as instruments and IV LASSO

	(1)	(2)	(3)	(4)
	OLS	IV with PDS-selected variables and full regressor set		
	All	All	No Mali	Mali
Child Height for Age z-scores				
Village Sanitation Coverage 20-39	0.019 (0.109)	−0.040 (0.262)	0.110 (0.276)	−0.410 (0.427)
Village Sanitation Coverage 40-59	0.104 (0.099)	0.259* (0.151)	0.389* (0.199)	0.246 (0.285)
Village Sanitation Coverage 60-79	0.163 (0.102)	0.281* (0.156)	0.200 (0.246)	0.165 (0.249)
Village Sanitation Coverage 80-100	0.194* (0.102)	0.280* (0.152)	0.371** (0.171)	0.163 (0.270)
Sample Size (individuals)	5481	5481	3063	2418
Sample Size (villages)	361	361	240	121
Control Mean	−1.818	−1.818	−1.885	−1.729
Control St. Dev.	1.290	1.290	1.236	1.355

Notes: This table reports the estimated effect of village sanitation coverage on child height for age z-scores from equation (5). Results are estimated using pooled samples of children under 5 at baseline for India, Indonesia, and Mali. We allow the impact of treatment to vary with baseline sanitation coverage by including indicator variables for quintiles of village sanitation coverage (with 0–20% being the omitted variable). Column 1 reports results from the OLS specification and columns 2–4 report results from the IV LASSO specifications. The set of instruments for village sanitation coverage are: treatment in each country, sanitation coverage at baseline at the village level (quartiles), and sanitation coverage at baseline (quartiles) interacted with treatment and country dummies. Column 2 selects the following instruments for Panel B: treatment in Mali, sanitation coverage at basline at the village level (quartiles), and sanitation coverage at baseline (quartiles) interacted with treatment and Indonesia country dummy. Column 3 selects the following instruments for Panel B: sanitation coverage at basline at the village level (quartiles). Column 4 uses the following instruments for Panel B: treatment in Mali, sanitation coverage at basline at the village level (quartiles), and sanitation coverage at baseline (quartiles) interacted with treatment and Mali country dummy. Column 1 includes controls for baseline height of the child, country, and an indicator of the randomization block and includes a separate set of controls all measured at baseline for each country interacted with a country dummy. The Indonesia controls include child age and sex dummies, education of the household head, household size, household per capita income, dirt floor, village is within a 10-min walk from a river, and discount rate. The India controls include age and sex dummies, improved water source, hand washing station with soap and water, caregiver had correct knowledge about diarrhea, caregiver had correct knowledge about risks of open defecation, age and sex of household head, household size, and household income. The Mali controls include child age and sex dummies, education and language spoken by the household head, OD disapproval, asset index, and social capital index. The lasso-selected controls vary in columns 2–4. Robust standard errors are clustered at the village level and are reported in brackets below the main effects. ***p < 0.01, **p < 0.05, *p < 0.1.

**Table A8:** Impact of Treatment on Open Defecation (0/1)

	(1)	(2)	(3)
	Full Sample	Households with Private Sanitation at Baseline	Households without Private Sanitation at Baseline
Indonesia			
Treatment	−0.029 (0.029)	−0.035 (0.028)	−0.064** (0.030)
Sample Size	1903	966	937
Control Mean	0.519	0.240	0.826
India			
Treatment	−0.051* (0.027)	−0.043 (0.089)	−0.050*** (0.016)
Sample Size	1655	202	1453
Control Mean	0.911	0.427	0.974
Mali			
Treatment	−0.363*** (0.035)	−0.373*** (0.042)	−0.360*** (0.036)
Sample Size	3981	1383	2598
Control Mean	0.855	0.744	0.916
Tanzania			
Treatment	−0.125*** [0.028]	−0.007 [0.006]	−0.135*** [0.034]
Sample Size	1786	467	1319
Control Mean	0.233	0.005	0.299

Notes: This table reports the estimated effect of treatment on the household's degree of open defecation from equation (7). Each panel represents a different sample and each column a different specification. Each treatment effect comes from a separate linear regression. See tables in the appendix for baseline balance results. Indonesia regressions include sub-district fixed effects, India block fixed effects, Mali district fixed effects, and Tanzania district fixed effects. Robust standard errors are clustered at the village level in Indonesia, India, Mali and Tanzania; these are reported in brackets below the treatment effects. ***p < 0.01, **p < 0.05, *p < 0.1.

## Data Availability

Data will be made available on request.

## Appendix Experimental Design and Data

### Sample Selection and Randomization

The data used in this paper were generated from cluster-randomized interventions in all four countries. The unit of randomization was the village in all four countries. In 3 out of the 4 countries, the villages were first clustered into strata and then the villages were randomized into treatment and control groups within each stratum. The data used are from baseline and follow-up surveys of a random sample of households with children under 2 years of age at baseline. In general, the samples for all four countries appear to be well balanced at baseline, have low levels of attrition, and show little evidence of attrition bias. The details of the random assignment and data collection are presented in Table 1 and discussed in the data appendix.

The evaluation occurred in eight districts of rural East Java, Indonesia; in 2 districts in Madhya Pradesh, India; the region of Koulikoro in Mali, and 10 districts in Tanzania. The strata used for random assignment are sub-districts in Indonesia, blocks in India, and districts in Tanzania. There were no stratifications used in Mali. Detailed information on sample selection and randomization can be found for each country in Cameron and Shah (2010), Cameron et al. (2013), Patil et al. (2013), Alzua et al. (2014), and Briceño et al. (2017).

In Indonesia, during the study period, trained facilitators went into the eight project districts and supported the local governments in conducting triggering and follow-up activities in the communities. Of the 80 treatment villages, the endline survey data shows that 53 villages (66 percent) were triggered.^16^ Approximately 14% of control villages were triggered. Initial sample size calculations indicate that this level of non-compliance was not sufficient to under power our results.

In India, two districts in Madhya Pradesh participated in the impact evaluation of TSC (Dhar and Khargone), for a total of 80 rural villages. One hundred percent of the treatment villages received the program. Based on interviews with block level Total Sanitation Campaign (TSC) officers, all treatment GPs received at least some funds with more than half receiving 100% of the allocated budget for TSC. Block officers also reported that 10 of the 40 control villages (∼25%) received the TSC program.

In the case of Mali, 100% of the treatment communities received the program and 59 out of 60 communities achieved Open Defecation Free Certification. While 10% of the control communities reported to have received some program for constructing latrines, they do not mention Unicef or the Malian Government. The team was not aware of any other NGOs promoting sanitation in the region of the study at that time, but some local churches and advocacy groups may be present to promote improved sanitation.

Tanzania is administratively separated into 30 Regions, 169 Districts and 3643 Wards, with the average ward holding approximately 12,000 people. The sample was drawn from 10 districts spread throughout the country selected by the Ministry of Water (MoW) and Ministry of Health and Social Welfare (MoHSW) to provide geographic diversity at the national level. To evaluate the impact of TSSM, a cluster-randomized evaluation with random assignment of interventions at the ward level was implemented (including 44 treatment and 46 control wards). Wards were identified as the optimal operational unit of implementation for the project, and of sufficient geographic extension to minimize the risk of significant information spillovers between populations exposed to the localized messages, community events, and other forms of social promotion activities. Program reports suggest that 86% of wards were triggered with TSSM activities and that there was no contamination into control areas.

### Data Collection

The data collection efforts focused on households with children under 2 years of age as young children's health is affected the most by poor sanitation (Murray and Lopez, 1997). All countries used similar and standardized structured questionnaires, but questionnaires were also modified to suit the local research needs. The survey collected data on social and demographic characteristics of the household and its members, information on household income, assets and labor force activity, household infrastructure and services for sanitation, hygiene and water, major housing facilities and amenities, and child school attendance and care. The survey also elicited the sanitation and hygiene knowledge, attitudes, and practices of the main caregivers of children under 5 years. The health survey recorded recent illness for children under 5 years and anthropometric measurements.

In Indonesia, the baseline survey was conducted in both treatment and control communities in August–September 2008. A total of 2087 households with 2353 children under 5 years in 160 sub-villages were interviewed. The sample frame of households with at least one child under 2 years was determined by lists provided by the community health care in each sub-village. Thirteen households were then randomly selected from this listing to participate in the baseline survey. The follow-up data collection was conducted approximately 24 months later, between November 2010 and February 2011. The endline survey used the same field methodology as the baseline survey. The final sample size in the endline survey included 1908 households from the baseline survey with no significant differential attrition between treatment and control groups.

In India, the baseline survey was conducted in May–July 2009. From the 80 villages, we completed surveys of 1954 households. The survey firm listed and mapped households in each village with information on whether they had a child under 2 years. From the list of eligible households, 25 households were randomly selected by the supervisor of the survey team using systematic random sampling. The follow up survey was conducted in February–April 2011–21 months after the baseline survey. The final sample size in the endline survey included 1655 households from the baseline survey with 2046 children, again with no significant differential attrition between the treatment and control groups.

For Mali, baseline information was collected between April and July 2011. A census was conducted in all 121 communities and all households with children under 10 were interviewed. A total of 4532 households were interviewed at baseline. The follow up survey took place between April and June 2013 and a total of 4031 households could be matched to the ones present in the baseline. Finally, children with complete anthropometric measures matched at baseline and follow up total approximately 2619.

Tanzania lacks a baseline survey and only had endline data. Although a baseline data collection was intended, unanticipated problems with the reliability of data resulted in the cancelation of field work in five out of the 10 districts originally planned and the impossibility of using the data to validate the randomized design, as it was originally planned. However, an endline survey was conducted in 2012. Sample size details are given in Table 1.

### Tests of Balance and Attrition

Randomization aims to minimize systematic differences between the control and treatment groups. However, the extent of sample attrition and the degree to which attrition is nonrandom is a legitimate concern in any follow-up survey. In India, of the 1954 households surveyed at the baseline, 299 were lost and 1655 were surveyed in the follow-up survey (15% attrition). The sample loss was not differential between the treatment (154) and control (145) groups. Patil et al. (2013) show that the attrition was also not differential by the treatment and control groups based on several important characteristics and that the baseline samples are well balanced between treatment and control villages. The few variables that were not balanced at the baseline are adjusted for in our analysis.

In Indonesia, of 2087 households interviewed at the baseline, 8.5 percent of households were lost and 1908 households were successfully re-interviewed in the follow up survey. The loss was similar in treatment and control villages; 86 households in the control and 93 households in the treatment group. Cameron and Shah (2010) show that the baseline sample was well balanced and Cameron et al. (2013) report that attrition did not result in significant imbalance in important household characteristics between treatment and control villages. The few variables that were imbalanced at the baseline are adjusted for in our analysis.

For Mali, 4532 households were present at baseline and 12.5% were either lost or could not be matched to the ones in the baseline. In the follow up, 5206 households were interviewed and 4031 were matched to baseline ones. The loss does not differ between treatment and control groups and that attrition did not produce an imbalance. (Alzua et al., 2014).

Tests of balance and attrition for Tanzania are challenging since there was no baseline survey. However, we show balance for a combination of time-invariant indicators and retrospective responses asked in the endline dating to February 2009, before the intervention had started. Table A4 suggests fairly good balance. Differential attrition due to migration or other causes could also be a concern. However, data from the complete census listings of selected enumerator areas provide evidence of limited migration and attrition. Less than 5% of households moved into the community within the three-year intervention period, and this does not differ across treatment/control groups. We mitigate any confounding that migration may cause by restricting the sample to households residing in the area since 2009.

## Footnotes

2 Several observational studies of interventions that prevent human feces from entering the environment have been shown to reduce diarrheal disease (Clasen et al., 2010; Norman et al., 2010), enteric parasite infections (Barreto et al., 2010, Ziegelbauer and Speich, 2012) , and child stunting and mortality (Checkley et al., 2004, 2008; Humphrey 2009; Spears 2012).

3 All four of these CLTS interventions have published impact evaluations for the individual country (see Alzua et al., 2014 for Mali, Cameron et al., 2019 for Indonesia; Briceño et al. (2017) for Tanzania, and Patil et al., 2013 for India). In this paper we provide more global evidence focused on community coverage of sanitation facilities.

4 In some IV specifications we exclude Mali because our exclusion restriction requires that the program only operates through increasing village sanitation. This may be violated in Mali due to other behavioral change impacts e.g. on handwashing behavior.

5 https://www.communityledtotalsanitation.org/page/clts-approach.

6 Such shaming could result in negative impacts on poorer households who may not be able to afford to build their own toilet. We are not aware of any research examining such impacts directly. Cameron et al. (2019) find that CLTS reduces toilet construction in treatment communities with very low levels of social capital, possibly reflecting a backlash against such shaming in communities where there are low levels of social cohesion.

7 GDP per capita in US PPP dollars by country in 2020: Indonesia – 11,445; India – 6118; Tanzania – 2635; Mali – 2217 (World Bank). Human Development Indices by country in 2019: East Java, Indonesia – 0.717; Madhya Pradesh, India – 0.603; Tanzania – 0.529; Koulikoro region in Mali – 0.431 (UNDP Human Development Report).

8 As our samples consist of households with children under the age of 2 at baseline, our village sanitation coverage variable is the percentage of these families who have access to sanitation. Data for Indonesia (the 2014 Indonesian Family Life Survey) allow us to examine whether this is a good proxy for sanitation coverage in the village as a whole and suggest that it is. The two measures are highly correlated (correlation coefficient of 0.93).

9 The WHO reference group consist of 8440 healthy breastfed infants and young children from diverse ethnic backgrounds and cultural settings (Brazil, Ghana, India, Norway, Oman and USA). See WHO Multicentre Growth Reference Study Group (2006).

10 Cameron et al. (2019), Pickering et al. (2019), Null et al. (2018)Luby et al., 2018 Clasen et al. (2014), Patil et al. (2014) and Briceño et al. (2017) report results from randomized controlled trials of sanitation interventions in Indonesia, Bangladesh, Kenya, Zimbabwe, various states in India, and Tanzania and find no effect on child height. In contrast, Dickinson et al. find that CLTS treatment was associated with 0.37–0.52 standard deviation increase in HAZ in Orissa India; Hammer and Spears (2016) and Cameron et al. (2021) find increases in height as a result of improved village sanitation resulting from India's TSC in rural Maharashtra; and CLTS with added financial incentives in Laos, respectively.

11 Meager (2019) and Vivalt (2020) use Bayesian Hierarchical models to pool results across randomized controlled trials. Applying these methods, particularly when using instrumental variables methods, is non-trivial and beyond the scope of this paper. These methods are worthy of exploration in future research in this area.

12 The IV estimates of the effect of village sanitation on height are larger in magnitude than the OLS estimates (Panel A), although with overlapping 95% confidence intervals.

13 Table A7 in the appendix presents results where we allow for thresholds at each quintile of the sanitation coverage distribution. It detects a similar, but less well-fitted patterns.

14 The samples are balanced across control and treatment groups for these sub-samples (Appendix Tables A1-A4).

15 In Tanzania the dependent variable is an indicator variable equal to 1 if the household reported that householders usually defecate in the open, and 0 otherwise. Table A8 in the appendix presents results when open defecation is defined using an indicator variable for all countries. The results are similar to those in Table 7 when the OD intensity index is used.
